# Reflector variables in augmented reality lineups: Assessing eyewitness identification reliability in children and adults with confidence, response time, and proximity to the lineup

**DOI:** 10.1371/journal.pone.0308757

**Published:** 2024-09-18

**Authors:** Heather L. Price, Ryan J. Fitzgerald

**Affiliations:** 1 Thompson Rivers University, Kamloops, Canada; 2 Simon Fraser University, Burnaby, Canada; University of Glasgow, UNITED KINGDOM OF GREAT BRITAIN AND NORTHERN IRELAND

## Abstract

Attending to the behaviors of eyewitnesses at police lineups could help to determine whether an eyewitness identification is accurate or mistaken. Eyewitness identification decision processes were explored using augmented reality holograms. Children (*n* = 143; *M*_age_ = 10.79, *SD* = 1.12 years) and adults (*n* = 152; *M*_age_ = 22.12, *SD* = 7.47 years) viewed staged crime videos and made identification decisions from sequential lineups. The lineups were presented in augmented reality. Children were less accurate than adults on the lineup task. For adults, fast response times and high post-identification confidence ratings were both reflective of identification accuracy. Fast response times were also reflective of accuracy for children; however, children’s confidence ratings did not reflect the likely accuracy of their identifications. A new additional measure, the witness’ proximity to the augmented reality lineup, revealed that children who made mistaken identifications moved closer to the lineup than children who correctly identified the person from the crime video. Adults who moved any distance towards the lineup were less accurate than adults who did not move at all, but beyond that, adults’ proximity to the lineup was not reflective of accuracy. The findings give further evidence that behavioral indicators of deliberation and information-seeking by eyewitnesses are signals of low lineup identification reliability. The findings also suggest that when assessing the reliability of children’s lineup identifications, behavioral measures are more useful than metacognitive reports.

## Introduction

Recent advances in eyewitness identification lineup administration have led to a better understanding of witness decision-making. Beyond the basic “*Is he there*?” decision, researchers have assessed judgments of witness confidence, used confidence assessments to indirectly infer witness decisions, and employed technology like eyetrackers to understand how visual behavior relates to decision accuracy. In the present work, we investigated witness lineup behaviors using a new method of presenting eyewitnesses with sequential lineup stimuli that tracks witness behavior: augmented reality holograms.

Augmented reality differs from virtual reality in that a holographic image is inserted into an existing environment, allowing the wearer to remain visually present in their environment. This contrasts with virtual reality, where the user loses visual access to their real-world surroundings. The lack of immersion in augmented reality comes with several benefits, including a lack of some challenges posed by immersion for some users (e.g., dizziness, nausea) and increased safe portability. As a result, augmented reality provides an ideal medium for privately viewing images.

Here, we explore the use of augmented reality lineups in both children and adults. Though there has been substantial evidence that young adults outperform children on eyewitness identification tasks, there is also growing evidence that children can perform better than previously thought [1]. Thus, in the present work, we compared children and young adults to further explore their differences and similarities in lineup identification accuracy.

### Extra-identification decision information

Eyewitness identifications are categorical recognition decisions (there/not there), which tells us little about *why* the decision was made. Efforts to contextualize the identification decision often focus on witness confidence assessments that take place immediately after the decision and apply to the lineup decision (i.e., confidence in selection/rejection). Under ideal lineup conditions, such as in controlled experiments that involve no biasing procedures, confidence increases are associated with increases in suspect identification accuracy [2]. The lineup procedure has also been innovated to collect judgments about the likelihood that each individual lineup member is the target [3–5], which allows for more information about witness memory to be extracted. Confidence statements and all other behaviors during the lineup procedure that co-vary with identification accuracy are known as *reflector variables* because they reflect whether the suspect in the lineup is guilty or innocent [6].

Information-seeking behaviors may indicate that the eyewitness has a poor memory and reflect an increased risk of mistaken identification. Support for the inverse relationship between information-seeking and accuracy has been found in eyetracking studies [7–9]. This research shows that when the witnesses are visually navigating a lineup, correct identifications are associated with fewer comparisons across lineup members than incorrect identifications [8,9]. However, Flowe [7] used eyetracking to explore differences in the search approach between simultaneous and sequential lineups and found that participants viewing sequential lineups spent more time analyzing foil images than those viewing simultaneous lineups, which Flowe suggested might be a result of more thorough visual processing promoted by individual image presentation.

Researchers have also explored measures that lead to inferences about the automaticity of a lineup decision, which itself is linked to underlying memory strength for the target [10,11]. For instance, Charman and Cahill [12] found that post-lineup memory for non-target lineup members (i.e., fillers) was inversely related to identification accuracy. The implication is that witnesses with weaker memories of the target are less likely to experience automatic recognition when examining the lineup members, which results in more time spent looking at fillers and, in turn, stronger memories for those fillers.

Decision latency is another common proxy of automaticity. The signal detection model of recognition memory suggests that decision time indexes the degree of match between a test stimulus and memory for a previously viewed stimulus [e.g., 13]. Quicker decision times are associated with a less deliberative decision process and thus, the inference is made that quicker decisions may be indicative of stronger memory for the target [e.g., 14]. Indeed, several studies have shown that faster decision times are related to greater accuracy among witnesses who choose from a lineup [14–20]. Note, however, that most of the decision time research has been conducted with simultaneous lineups.

The relation between latency and accuracy for sequential lineup decisions is less well understood. Despite the many sequential lineup studies published since the procedure was first introduced [21], decision latency is reported in only a few papers and the literature is less conclusive on whether correct identifications from sequential lineups are faster than incorrect identifications. Wilson et al. [22] reported that sequential lineup hits were made faster (5 s) than false alarms (7 s). Sporer [23] also found a latency-accuracy association for sequential lineups. However, other studies have not observed a similar relationship [e.g., 7].

There are theoretical reasons why latency might not relate to sequential lineup decision accuracy. Sequential presentation minimizes the opportunity to compare lineup members, which could disrupt the latency-accuracy relation [20]. Further, the speed associated with accurate identifications has been explained as a ‘pop-out’ effect, such that one lineup member is automatically recognized and the others are immediately discounted [16]. Although the pop-out phenomenology is not fully understood, if the effect is reflective of a perceptual distinction between one lineup member and the others (e.g., increased fluency) it could be diminished by presenting each lineup member in isolation. A target would, in effect, have no others from whom to pop out. Nevertheless, in addition to the latency-accuracy associations reported in the lab research [3,23], pop-out effects with sequential lineups were reported in a field study with eyewitness of real crimes [24]. However, with field data the accuracy of suspect identifications cannot be determined. Further lab research is needed to determine whether fast identifications are more accurate than slow identifications from sequential lineups.

### Child vs. adult eyewitnesses

Young adults typically outperform children in eyewitness identification experiments, but reflector variables might nonetheless be useful for determining when eyewitness identifications by children are reliable. Children are more likely than adults to make a mistaken identification in culprit-absent lineups and are also less likely than adults to correctly identify the culprit in culprit-present lineups [25–27]. In a large study comparing the metacognitive abilities of children (10 to 13-years-old, *n* = 619) and adults (Mean age = 24, *n* = 600), Keast et al. [28] found that contrary to adults, whose confidence ratings were well calibrated with accuracy, children’s confidence ratings often did not reflect accuracy. Across the two lineups tested in their first experiment, for instance, suspect identification accuracy ranged from 30–60% for children who were 90–100% confident. Winsor et al. [1] note, however, that the suspect identification accuracy rates reported by Keast et al. [28] were not corrected for the likelihood that some mistaken identifications would be siphoned away by known-innocent lineup fillers.

One way to account for fillers is by dividing the overall number of mistaken identifications in the culprit-absent lineup by the nominal size of the lineup (i.e., the number of lineup members). Winsor et al. [1] used this nominal lineup size correction to recompute suspect identification accuracy rates for the sample of child witnesses studied by Keast et al. [28]. After applying the nominal size correction, Winsor et al. estimated a suspect identification accuracy rate of 86% for children with 90–100% confidence. Children have also been found to have the metacognitive ability to provide confidence ratings for all the lineup members, resulting in accuracy rates similar to those for children asked to report a single categorical identification decision [29,30]. Winsor et al. also tested a new interactive lineup with children, which could be rotated to see different angles of the lineup members (see also [31]) and found that suspect identification accuracy was negatively associated with the length of time interacting with the lineup members. This association provides evidence from child witnesses that information-seeking behaviors during the lineup procedure are indicative of a weak memory trace and low identification reliability.

## Present research

In the present work, we explored a new lineup medium that we anticipate will be particularly useful to researchers looking to better understand eyewitness identification decisions. We developed a sequential holographic lineup that is administered using augmented reality technology (i.e., *Microsoft HoloLens*). Holographic images of people were scanned in to the virtual environment and then presented as 3D images that can be viewed from a distance, as in a typical lineup, but can also be approached and explored in three dimensions (i.e., a witness can walk around the hologram), and these movements can all be measured and recorded. Thus, the information that can be obtained from holographic presentations exceeds that which can be obtained by other means. Further, holographic lineup administration is naturally blinded because the images are viewed through the goggles and the administrator does not know which lineup member is being viewed at any given time. Presentation of images can be randomized which requires an administrator to look up the identifying number after the lineup is complete, thus further reducing any chance of bias.

Holographic images are excellent quality representations of real people which can be approached closely (participants can get as close as 85cm before the image is lost). Thus, they may provide a viable method for presenting the types of cues available at a live lineup without the practical drawbacks and stress that witnesses report at live procedures [32]. Further, witness anonymity can be assured, unlike in some live lineups. Though whether a holographic lineup would reduce stress compared to a live lineup is an empirical question, witnesses tend to feel more threatened and socially awkward when looking at people in a live lineup [33]. Holographic lineups are also easily editable (e.g., to allow clothing standardization). Finally, though in the present study we opted to conduct all lineups in the laboratory, a witness could be returned to the scene of the crime and don the goggles to be physically reinstated in the context during the identification task (see [34] for a similar discussion of benefits of virtual reality with virtual busts).

This study, the first to our knowledge with holographic lineups, was an exploratory investigation of witness behavior observed from sequential holographic lineups, and the potential relations between such behavior and identification accuracy. Though prior work with eyetrackers and 3D models [7,31,34] provide some direction for hypothesis development in simultaneous lineups (e.g., longer looking time for incorrect identifications, fewer lineup member comparisons for correct than incorrect identifications, longer looks at suspects than foils), there are important differences in the information obtained by augmented reality versus a static image lineup. With augmented reality, we are able to measure 3D interaction with the image (i.e., including up-close physical inspection). Thus, though we were guided by the prior literature, this was primarily an exploratory study.

Though we hypothesized the adults would generally outperform children, we also anticipated that both age groups would show similar patterns. We hypothesized that witnesses who made quicker decisions [14] and witnesses who self-rated decision confidence as high [2] would be more likely to be accurate. For proximity (i.e., the amount the participants moved toward the lineup), there is no previous research to directly inform our hypotheses, but approaching the image more closely could be a form of information-seeking behavior and thus, be reflective of a deliberative process. This could be expected to result in a negative association between identification accuracy and the participant’s physical proximity to the lineup. However, given the unique nature of the stimulus, even a witness with a strong memory for the culprit might also approach the images closely.

## Method

### Development and testing

Our initial testing of the technology involved trialing several options for scanning people into the software as holograms. Images for the present study were scanned using a commercially available scanning application (https://itseez3d.com/). Images of the busts of real people were scanned, with minor image editing to smooth out unnatural image components (e.g., often related to hair).

A computer programmer was hired to program into the holographic lineup measurements of how long the witness looked at each image, how many times the witness looked at each image, and how closely the witness approached each image (on the x, y, and z axes). Further, the HoloLens recorded a high-quality digital video taken from the perspective of the witness. Thus, an evaluator of the lineup or witness can subsequently review the footage and experience the lineup exactly as the witness did.

To ensure familiarity with the HoloLens prior to data collection, we provided practice to participants in using the HoloLens prior to the target lineups. We developed a practice lineup (with cartoon animals), which provided participants guided practice in navigating the augmented reality environment. Participants were talked through how to switch between images, how to move physically towards the images, and how to ‘end’ a lineup (i.e., to make a decision). Initial tests indicated that participants easily grasped the navigation of the lineups (programmed to be navigated by voice, remote, or gesture). Participants were also comfortable moving about the physical space in order to view the holographic image from multiple angles.

### Participants and design

The initial sample comprised 154 undergraduate students (*M*_age_ = 22.12, *SD* = 7.47 years; *n* = 104 males, 50 females) and 143 children (*M*_age_ = 10.79, *SD* = 1.12, range = 8–13 years; *n* = 101 males, 42 females). Undergraduate students participated in the experiment for course credit. Children were recruited from a technology summer camp and received a small prize for their participation. This study was a 2 (child vs adult witness) x 2 (culprit-present vs culprit-absent lineup) design. Participants individually viewed two culprit videos (one male, one female culprit and one culprit-absent, one culprit-present lineup; all randomly assigned), and made two identification decisions from sequential lineups. Due to technical problems, all data from two adult participants and one trial from a third adult participant were excluded, resulting in a final sample of 152 adults (and total of 303 lineup decisions for adults). There were no exclusions in the child sample, which included a total of 286 lineup decisions. Data were collected from November 9, 2017-August 20, 2018. This research was approved by the Thompson Rivers University institutional research ethics board (#101665). This study was not pre-registered. Data are available on the Open Science Framework: https://osf.io/rfje9/.

### Videos and lineups

Culprit presence was manipulated by the crime video viewed, rather than by the lineup presented [35]. Thirty-two culprit videos were used, each with the same basic actions, but each featuring a different actor. The crime videos were obtained from the Eyewitness Lineup Identity database [36]. Each video is approximately 25–30 seconds in duration and depicts a single culprit stealing a laptop from a bag left in a hallway. The culprit was in view for approximately 22 seconds. Sixteen of the videos featured a female culprit; 16 of the videos featured a male culprit. The lineup images were recorded on the same day as the crime videos. All people in the images for a lineup were matched on race, approximate age, and eye and hair color. No accessories (e.g., eyeglasses) were present and all men were clean-shaven. Images of 16 of the culprits were used to create two 8-member lineups (one with men and the other with women). Images from the other 16 videos were never present in the lineups (8 men, 8 women). The presence of the culprit depended on whether the crime video depicted one of the culprits who was included in a lineup (culprit-present) or one of the culprits who was not included in the lineups (culprit-absent). The order in which images were presented in the lineup was randomized across participants, with the exception that the culprit was never presented first.

### Reflector variables

#### Confidence

After providing their lineup decision, participants rated their decision confidence on a scale from 0% (uncertain) to 100% (confident).

#### Response time (RT)

RT is the time taken to respond to the lineup task, measured from presentation of the lineup to the participant’s identification or nonidentification response.

#### Proximity

Distance from the HoloLens camera to the image is separable along x (horizontal distance, walking sideways from the image), y (vertical distance, jumping or otherwise gaining or losing height), and z (distance in front of the HoloLens, walking towards or away from the image) axes. Images were always initially presented at eye height (i.e., height adjusted for each participant) along the y-axis and directly in the center of the x-axis. For our measure of proximity we recorded the closest three-dimensional distance to the lineup image, which incorporated all three axes (x, y, and z). Measures of proximity were polled every 0.25 seconds while the participant explored the lineup. Participants began the experiment at a distance of 4m (along the z-axis) from the images, and thus raw proximity scores could range from a minimum 4 m from the lineup (i.e., no movement) to maximum score of 0 m from the lineup. When analyzing proximity, the values were reverse-scored by subtracting from 4. Thus, after reverse scoring, a value of 0 indicates no movement with scores increasing as participants moved closer to the lineup.

### Procedure

After providing written consent from adult participants and verbal assent from child participants (in addition to parental consent), participants viewed a video (video culprit gender order was counterbalanced) and received the following instructions: “*You are about to watch a brief video*. *Please pay close attention to the video and then I will ask you some questions after it*.*”* Immediately after the first video, the second video was played of the culprit of a different gender (instructions: “*Now I will show you one more video*. *Please pay close attention again*.*”)*. After viewing the two videos, participants completed a demographics form and a questionnaire about prior exposure to augmented and virtual reality technology. Participants were then taken to a second room and were introduced to the augmented reality goggles. Participants then received an introduction to the goggles, which were adjustable to each participants’ head size, and practice in navigating the augmented environment with the cartoon animal lineup. Once participants were able to accurately follow basic instructions with the practice lineup, the lineup was launched. The first lineup was always the lineup for the first video watched. Participants were given the following instructions:

*“Now, you will see several images of people. These images are digital scans of real people. It is important for you to know that the person from the video may or may not be in the images. It is also important for you to know that I don’t know which people you are seeing or if the decision you make is correct. Once you have looked through all the images as much as you like, I would like you to tell me if you think the person is there, and if so, which number the person is. You will have to look at all of the images at least once before you are able to make a decision. However, you can go back and forth between images as often as you’d like before you make a decision.”*

Participants were able to navigate the lineup as they wished (e.g., going back and forth between images), with the restriction that they could not make an identification decision before viewing all images. Participants were not required to complete a full lap prior to free navigation. Participants were instructed to click “end lineup” when ready to make a decision, thus stopping the timer. After providing their lineup decision, participants provided a confidence rating.

After a short break in which the research assistant loaded the next lineup, participants completed the lineup for the second culprit in the same manner as the first. Participants then completed a final questionnaire about ease of use of the goggles and whether they believed they would be better able to identify a culprit from augmented reality holograms. Most adults anticipated that it would be easier with augmented reality (74.8%) than with photographs (21.9%), with open-ended explanations often focusing on the additional information provided in augmented reality, relative to photographs, as the primary consideration in their selection. By contrast, most children anticipated it would be easier with photographs 58.0% (vs. 38.5% anticipating augmented reality would be easier). Children often could not explain why, but some stated that photos would be clearer.

#### Analysis

We performed analyses on the full sample and on a subgroup of responses classified as suspect identifications. For suspect identifications, we restricted the analysis to identification responses that were classified as hits or false alarms. Hits refer to correct identification of the culprit from a culprit-present lineup, which corresponds with a criminal investigation in which an eyewitness identifies a guilty suspect. All other responses to culprit-present lineups (filler identifications and nonidentifications) were excluded for the suspect identification subgroup analysis.

False alarms refer to mistaken identifications of innocent suspects from culprit-absent lineups, which were estimated via no correction, nominal size correction, and effective size correction. *No correction* classifies every mistaken identification from a culprit-absent lineup as a false alarm. This assumes every lineup member is an innocent suspect. The *nominal size correction* divides total mistaken identifications in culprit-absent lineups by the number of lineup members (i.e., 8). This assumes only one lineup member is a suspect and the lineup is perfectly fair. The *effective size correction* divides total mistaken identifications in culprit-absent lineups by the number of plausible lineup members [37], which is known as the lineup’s effective size [38]. Rather than assuming the lineup is perfectly fair, the effective size correction takes a measure of the number of plausible lineup members and assumes the innocent suspect would be one of them [39]. We calculated effective size using Tredoux’s [40] formula and measured it directly from the distribution of mistaken IDs in the culprit-absent lineup, as recommended by Quigley-McBride and Wells [41].

## Results

### Full sample

Participants spent an average of approximately one minute in the augmented lineup (Adult: *M* = 59.7 s, *SD* = 33.1; Child: *M* = 58.4, *SD* = 32.6). Across the eight lineup images, this corresponded with an average of 19.0 (*SD* = 8.2) image views for adults and 15.6 (*SD* = 7.9) image views for children.

Identification response probabilities for culprit-present and culprit-absent lineups are reported in Table 1. Adults were more likely than children to correctly identify the culprit in culprit-present lineups, *z* = 4.27, *p* < .001. Children were more likely than adults to mistakenly identify a filler in both culprit-absent lineups, *z* = 3.51, *p* < .001, and culprit-present lineups, *z* = 3.94, *p* < .001. Odds ratios (*OR*) are reported in Table 1. Using Cohen’s [42] conventions, *OR*s of 1.44, 2.48, and 4.27 are interpreted as small, medium, and large effect sizes [43]. Accordingly, for both correct identifications and filler identifications, the differences between the child and adult samples can be interpreted as medium in magnitude. The only nonsignificant difference between adults and children was in non-IDs in culprit-present lineups, *z* = 0.88, *p* = .377.

**Table 1:** Age differences in identification response.

		Adult			Child			Effect Size		
Lineup	Response	%	*SE*	*n*	%	*SE*	*n*	*OR*	*LL*	*UL*
Culprit-Present	Culprit ID	65.4	3.8	100	40.6	4.1	58	2.76	1.72	4.42
	Filler ID	19.6	3.2	30	40.6	4.1	58	2.80	1.66	4.71
	No ID	15.0	2.9	23	18.9	4.3	27	1.32	0.71	2.42
				153			143			
Culprit-Absent	Filler ID	51.3	4.1	77	71.3	3.8	102	2.36	1.45	3.83
	No ID	48.7	4.1	73	28.7	3.8	41	2.36	1.45	3.83
				150			143			

Note: *SE* = Standard Error, *OR* = Odds Ratio, *LL* = Lower Limit 95% CI, *UL* = Upper Limit 95% CI.

### Suspect identifications

For the analysis of suspect identifications, there were 177 identifications by adults (100 hits, 77 false alarms) and 160 identifications by children (58 hits, 102 false alarms). Suspect identification accuracy, otherwise known as positive predictive value [44], was computed by dividing the number of hits by the total number of suspect identifications (i.e., hits + false alarms). With no correction to the false alarm rate, suspect identification accuracy was greater for adults (56.5%) than for children (36.3%), *z* = 4.27, *p* < .001, *OR* = 2.28, 95% CI [1.47, 3.55]. With the nominal size correction, accuracy increased to 91.2% for adults and 82.0% for children, a nonsignificant difference, *z* = 1.72, *p* = .085, *OR* = 2.27, 95% CI [0.93, 5.58]. The average effective size of the lineups was 5.65 for children and 4.64 for adults (Table 2). With the effective size correction, accuracy was 85.8% for adults and 76.3% for children, a nonsignificant difference, *z* = 1.61, *p* = .106, *OR* = 1.88, 95% CI [0.89, 3.94].

**Table 2:** Effective size of lineups, estimated from distribution of culprit-absent lineup choices.

		Culprit-Absent Lineup Choices								Effective Size		
Age	Lineup	LM1	LM2	LM3	LM4	LM5	LM6	LM7	LM8	*E*	*LL*	*UL*
Child	A	8	1	6	4	11	13	2	13	5.80	4.91	7.09
	B	3	7	1	2	10	11	2	8	5.50	4.48	7.11
Adult	A	5	1	1	1	12	7	3	10	4.85	3.83	6.61
	B	1	4	0	0	11	11	5	5	4.43	3.54	5.93

Note. LM = Lineup Member.

#### Confidence

A 2 (age: child vs adult) x 2 (accuracy: hit vs false alarm) ANOVA on confidence ratings resulted in a significant main effect of accuracy, *F*(1, 333) = 12.16, *p* < .001, *η*_*p*_*^2^* = .035, and a significant age-accuracy interaction, *F*(1, 333) = 8.01, *p* = .005, *η*_*p*_*^2^* = .023. Table 3 shows adults were more confident for hits than false alarms, with a large effect size according to Cohen’s [42] conventions; by contrast, children were almost no more confident for hits than for false alarms, with an effect size close to zero. The effect of age on confidence did not exceed the significance threshold, *F*(1, 333) = 3.46, *p* = .064, *η*_*p*_*^2^* = .010.

**Table 3:** Confidence, RT, and proximity to lineup for suspect identifications.

		Hits		False Alarms		Effect Size		
Measure	Age	*M*	*SE*	*M*	*SE*	*d*	*LL*	*UL*
Confidence	Child	71.3	2.8	69.9	2.0	0.07	-0.26	0.39
	Adult	81.2	1.6	67.8	2.0	0.78	0.45	1.10
	Total	77.6	1.5	69.0	1.5	0.45	0.23	0.67
RT (s)	Child	52.7	3.6	66.0	3.7	0.39	0.07	0.72
	Adult	50.9	4.3	71.5	4.3	0.64	0.32	0.95
	Total	51.5	2.2	68.4	2.8	0.51	0.29	0.73
Proximity (m)	Child	1.0	0.1	1.5	0.1	0.43	0.11	0.76
	Adult	0.6	0.1	0.9	0.1	0.22	-0.08	0.52
	Total	0.8	0.1	1.2	0.1	0.41	0.19	0.63

Note. Adults made 177 suspect identifications (100 hits, 77 false alarms) and children made 160 suspect identifications (58 hits, 102 false alarms). RT values represent the time taken to complete the lineup. Proximity values represent the maximum distance (in m) a witness moved towards the hologram at any point in the lineup.

#### RT

A 2 (age: child vs adult) x 2 (accuracy: hit vs false alarm) ANOVA on lineup decision RT resulted in a significant main effect of accuracy, *F*(1, 333) = 21.07, *p* < .001, *η*_*p*_*^2^* = .059. Table 3 shows RT was slower for false alarms than for hits, with a medium effect size. The main effect of age was not significant, *F*(1, 333) = 0.24, *p* = .625, *η*_*p*_*^2^* = .001, and the age-accuracy interaction was also not significant, *F*(1, 333) = 0.96, *p* = .329, *η*_*p*_*^2^* = .003.

#### Proximity

A 2 (age: child vs adult) x 2 (accuracy: hit vs false alarm) ANOVA on the proximity measure resulted in a significant main effect of age, *F*(1, 333) = 16.53, *p* < .001, *η*_*p*_*^2^* = .047, and a significant main effect of accuracy, *F*(1, 333) = 9.04, *p* = .003, *η*_*p*_*^2^* = .026. The main effect of age was associated with a medium effect size, *d* = 0.45, 95% CI [0.23, 0.66], with children moving an average of 1.2 m (*SE* = 0.8) towards the lineup, resulting in greater proximity than adults, who moved an average of 0.8 m (*SE* = 0.8). For the main effect of accuracy, Table 3 shows witnessed moved closer to the lineup for false alarms than for hits, with a small-to-medium effect size. The age-accuracy interaction was not significant, *F*(1, 333) = 1.40, *p* = .237, *η*_*p*_*^2^* = .004.

### Developmental trends

There is great practical interest in understanding at what age children’s identification accuracy becomes more adult-like. Thus, to assess for age-related changes in identification responses, we divided the child sample into three age groups: 8–9, 10–11, and 12–13. To maximize sample sizes, we looked at developmental trends in the full sample rather than only for suspect identifications.

In culprit-present lineups, the correct identification rate increased with age (Table 4). Compared to 12–13 year olds, the correct identification rate was significantly reduced for both 10–11 year olds, *z* = 2.32, *p* = .021, *OR* = 2.55, 95% CI [1.14, 5.67], and 8–9 year olds, *z* = 2.74, *p* = .006, *OR* = 5.63, 95% CI [1.54, 20.52]. The difference between 8–9 and 10–11 year olds was nonsignificant, *z* = 1.34, *p* = .181, *OR* = 2.21, 95% CI [0.68, 7.22]. The difference between adults and 12–13 year olds was also nonsignificant, *z* = 0.60, *p* = .550, *OR* = 1.26, 95% CI [0.59, 2.67]. Table 4 shows that the lower correct identification rates for 8–9 and 10–11 year olds was associated with higher filler identification rates on culprit-present lineups, relative to 12–13 year olds and adults.

**Table 4:** Developmental Trends in Identification (ID) responses.

Age	Lineup	Response						*n*
		Culprit ID		Filler ID		No ID		
		%	*SE*	%	*SE*	%	*SE*	
8–9 yrs	Culprit-Present	21.1	9.9	52.6	12.1	26.3	10.7	17
	Culprit-Absent	—	—	70.6	10.5	29.4	10.5	19
10–11 yrs	Culprit-Present	37.1	5.3	47.2	5.5	15.7	4.0	83
	Culprit-Absent	—	—	69.9	4.9	30.1	4.9	89
12–13 yrs	Culprit-Present	60.0	7.5	17.1	5.7	22.9	6.4	43
	Culprit-Absent	—	—	74.4	7.4	25.6	7.4	35
Adult	Culprit-Present	65.4	3.9	19.6	3.2	15.0	2.9	150
	Culprit-Absent	—	—	51.3	4.0	48.7	4.0	153

In culprit-absent lineups, the filler identification rate was stable across the three groups of children (Table 4). No significant differences in culprit-absent responses were detected in pairwise comparisons between the child groups (all *p* > .50). Compared to adults, the filler identification rate for culprit-absent lineups was significantly increased for both 12–13 year olds, *z* = 2.69, *p* = .007, *OR* = 2.76, 95% CI [1.29, 5.87], and 10–11 year olds, *z* = 2.75, *p* = .006, *OR* = 2.20, 95% CI [1.25, 3.88]; the effect size was similar for the comparison between adults and 8–9 year olds, but the sample size for the child group was smaller and the difference was not significant, *z* = 1.51, *p* = .132, *OR* = 2.28, 95% CI [0.76, 6.78].

**Fig 1:**
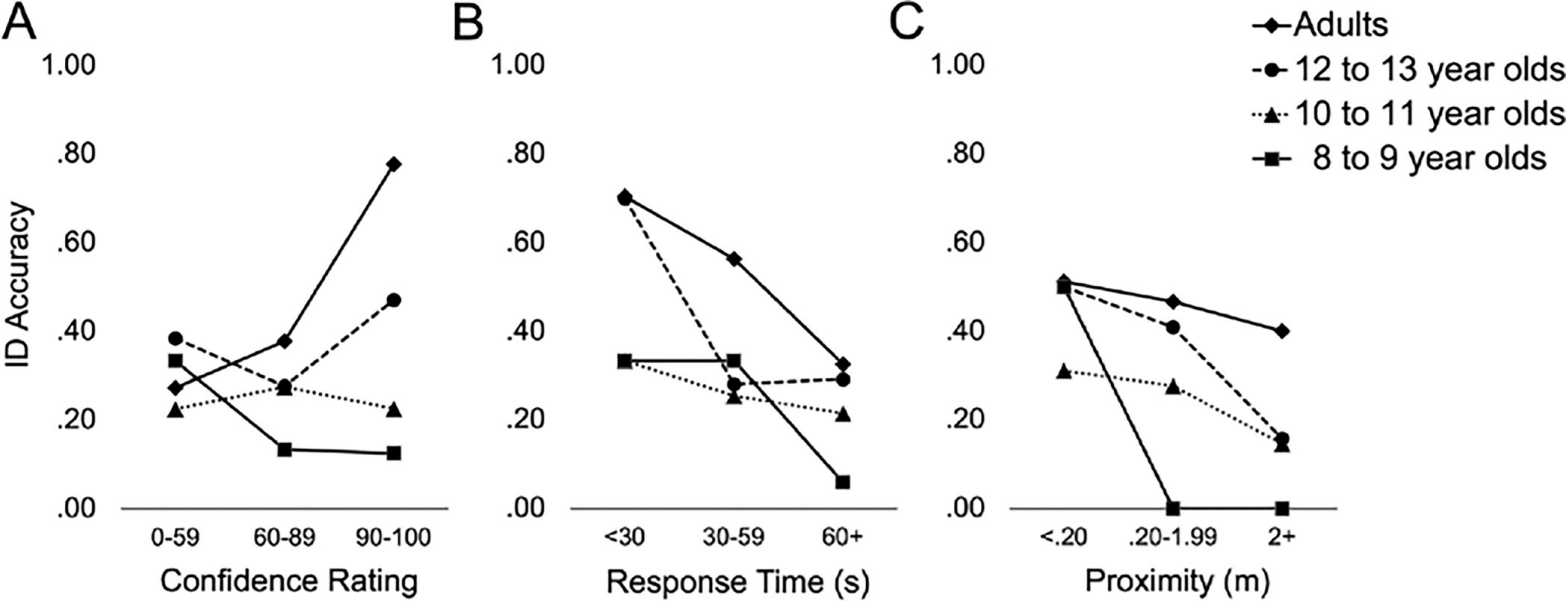
Developmental Trends in the Relation Between Accuracy and (A) Confidence, (B) Response Time, and (C) Proximity to the Lineup.

To assess for age-related improvements in meta-cognitive ability, we plotted accuracy rates at different levels of confidence ratings. For this analysis, we included any participant who made an identification. We included a small number of confidence bins to minimize the risk of small cell sizes; however, there were so few 8–9 year olds (*n* = 36 identifications, compared with 172 from 10–11 year-olds, and 78 from 12–13 year olds) that small cell sizes were inevitable for that group. Confidence-accuracy curves for adults and the three child groups are plotted in Panel A of Fig 1. Consistent with the suspect identification analyses, the curves show a stronger confidence-accuracy relation for adults than for children. In the highest confidence bin (90–100%), accuracy increased with age: 8–9 = 12.5% (*n* = 8), 10–11 = 22.6% (*n* = 31), 12–13 = 47.1% (*n* = 17), and adult = 77.8% (*n* = 63). In pairwise comparisons of accuracy rates for these high confidence identifications, there were no significant differences among the three child groups (all *p* > .07), and adults were significantly more accurate than all three of the child groups (all *p* < .02).

Fig 1 depicts accuracy rates for the age groups at different levels of RT and proximity (Panels B and C, respectively). A negative association between RT and accuracy was most apparent in the adult sample. For the child groups, accuracy was also negatively associated with RT; however, the strength of the association was less robust. In the fastest RT bin (< 30 s), accuracy for the 12–13 year olds (70.0%, *n* = 10) was almost identical to that of adults (70.4%, *n* = 27), *z* = 0.02, *p* = .983, *OR* = 1.02, 95% CI [0.21, 4.97]. Sample sizes in this bin were small for the child groups, however, and despite a numerical increase in accuracy for the 12–13 year olds over the two younger groups (both = 33%, combined *n* = 21), the difference did not exceed the significance threshold, *z* = 1.92, *p* = .055, *OR* = 4.67, 95% CI [0.92, 23.81]. The association between accuracy and proximity was also negative. For all three child groups, accuracy was lowest for those who moved the most toward the lineup (> 2 m) and highest for those who moved the least (< 0.20 m). The same trend occurred for adults, but the difference in accuracy between the two proximity extremes was less robust. Surprisingly, at the lowest proximity (< 0.20 m), the accuracy rate for 8–9 year olds (50.0%, *n* = 8) was as almost high as for adults (51.2%, *n* = 127). There were only eight identifications by 8–9 year olds in the low proximity bin, however, and there is a high risk of sampling error in the accuracy rate for that group. Nonetheless, it is notable that of the 16 identifications by 8–9 year olds who moved closer than 0.20 m toward the lineup, all the identifications were mistaken.

## Discussion

The novel application of augmented reality to sequential eyewitness identifications provided information about identification decisions that was not previously available. For adults, identification confidence and decision speed emerged as strong reflectors of whether an identified suspect was guilty or innocent. Decision speed also reflected accuracy for children, but confidence ratings had no meaningful relation to children’s identification accuracy. A new measure, proximity to the augmented reality lineup, was also reflective of accuracy in children: the less children moved toward the lineup, the more likely they were to be accurate.

Additional information-seeking behavior has been associated with deliberative decision processes and less automaticity [12,14]. In the present research, witnesses may have believed additional information was available by moving closer, seeking multiple angles, and studying the images for longer durations. However, these strategies did not improve performance. On the contrary, the witnesses who sought out additional information tended to be less accurate. This could indicate that information-seeking behaviors are interfering with successful recognition and resulting in poor performance. For instance, the best strategy might be to rely on the first impression when quickly scanning the frontal view of the lineup member, and any further attempts to improve upon that first impression by viewing from different angles might only confuse the witness and reduce performance. An alternative possibility, however, is that the information-seeking behaviors and poor performance were both caused by poor memory for the target [10,11].

In terms of meta-cognitive ability, the present findings are consistent with Keast et al.’s [28] conclusion that children have limited ability to communicate the likely accuracy of their identification with confidence ratings. We found that children were essentially just as confident in their false alarms as they were in their correction identifications. Furthermore, suspect identification accuracy for children who gave ratings of 90–100% confidence was virtually the same as for children who gave ratings of 0–59% confidence. When children were split into more fine-grained groupings, high confidence identifications were more accurate for older children (aged 12–13 years) than younger children (aged 8–11 years). Nonetheless, even high confidence identifications by the older children were much less accurate than those by adults.

One possible explanation for the weak confidence-accuracy relation in children is that the 100-point confidence scale was too complex. Keast et al. [28] pilot tested the same type of scale with children aged 10–14 and found that they could generate confidence ratings, find them on a scale, and translate verbal descriptions of quantities into corresponding confidence levels. When Keast et al. used the 100-point scale with an eyewitness identification task in their study, however, they concluded that children were overconfident and gave ratings that were poorly calibrated with accuracy. Other researchers have used much smaller scales (e.g., 5-point visual scale depicting cups of water with varying levels of fullness; [1,30]) and shown some success with children’s metacognitive abilities. Nonetheless, the present research provides further evidence that children are still developing their meta-cognitive skills, resulting in eyewitness identification confidence ratings that are less informative than what can be expected from adults.

Although children were generally less accurate than adults, this does not mean lineup identifications by child witnesses are inherently unreliable. Consistent with previous research [45], our study indicates that even though children’s meta-cognitive reports are often unrelated to their identification accuracy, objective behavioral measures can be used to diagnose the guilt of suspects who are identified by child witnesses. For instance, if fair lineups are assumed (i.e., by applying the nominal size correction), estimated suspect identification accuracy was 92% for children who made the decision in less than 30 s and 89% for children who moved less 0.10 m toward the lineup. By contrast, under the same assumptions (i.e., suspect identification from a fair lineup), estimated accuracy was reduced to 72% for children who took longer than 90 s to decide and 65% for children who moved more than 2 m toward the lineup. In summary, we identified two variables that reflect whether a suspect identified by a child witness is likely to be guilty or innocent. Furthermore, both variables are consistent with a general theory that lineup identifications are most likely to be reliable when the witness appears to instantly recognize someone in the lineup and does not seek additional information to aid the decision process.

### Limitations and future directions

As with much identification research, this work was conducted in a controlled laboratory environment, with participants witnessing an unemotional event. Limitations of laboratory experiments have been discussed many times before. However, the latter limitation is particularly important to consider here. Hologram realism may be a factor for witnesses who experienced encoding stress, as well as witnesses who experience stress during identification. Without emotional stimuli, we cannot know if hologram realism could negatively impact witnesses emotionally, and subsequently, their accuracy. Stress at encoding has been explored in a limited number of studies [46], but we know much less about stress at identification. Second, it is unclear if our results will extend to simultaneous lineups or lineups that are biased against an innocent suspect, both of which are common in the field.

Given the novelty of the augmented reality procedure, we made a number of procedural decisions, some of which may have impacted the present findings, and many of which could and should be explored another way. For example, participants were able to self-pace through the sequential procedure and the only restriction placed on participants’ lineup navigation was the requirement to view each image at least once prior to ending the lineup. We opted to allow for maximum freedom to understand how witnesses would naturally approach such a lineup. The lack of structure may decrease the likelihood that witnesses can focus quickly on initial reactions to lineup stimuli. However, given that much of the literature restricts witness behavior to a single, ordered lap through a lineup [e.g., 31], our methodological decision to allow witnesses control over the lineup permits greater comparing and contrasting between lineup members.

Finally, at this time, an important limitation is the cost and expertise required to purchase and develop a lineup in augmented reality. In the present experiment, there was extensive expertise required to program the lineup to collect particular types of data and interpret the resulting output. Currently, the level of expertise required will be well beyond the reach of most applied agencies. However, with increasing accessibility of technology, even smart phones often carry augmented reality technology, and thus, ease of use is likely to substantially improve.

## Conclusion

Continued consideration of extra-identification information will contribute to both a better understanding of eyewitness identification decisions and to the development of procedures that most fully inform investigators about likely witness accuracy. Here, we demonstrate that augmented reality lineups provide new information that can be used to contextualize eyewitness decisions. Consistent with many recent works, information-seeking behavior was a signal of low lineup identification reliability. This additional information will enhance our ability to distinguish accurate from inaccurate witnesses.

## References

[1] Winsor A. A., Flowe H. D., Seale-Carlisle T. M., Killeen I. M., Hett D., Jores T., et al. (2021). Child witness expressions of certainty are informative. *Journal of Experimental Psychology*: *General*, 150, 2387–2407. doi: 10.1037/xge0001049 34498905 PMC8721974

[2] Wixted J. T., & Wells G. L. (2017). The relationship between eyewitness confidence and identification accuracy: A new synthesis. *Psychological Science in the Public Interest*, 18, 10–65. doi: 10.1177/1529100616686966 28395650

[3] Sauer J. D., Brewer N., & Weber N. (2008). Multiple confidence estimates as indices of eyewitness memory. *Journal of Experimental Psychology*: *General*, 137, 528–547. doi: 10.1037/a0012712 18729714

[4] Sauer J. D., Brewer N., & Weber N. (2012a). Using confidence ratings to identify a target among foils. *Journal of Applied Research in Memory and Cognition*, 1, 80–88. doi: 10.1016/j.jarmac.2012.03.003

[5] Sauer J. D., Weber N., & Brewer N. (2012b). Using ecphoric confidence ratings to discriminate seen from unseen faces: The effects of retention interval and distinctiveness. *Psychonomic Bulletin & Review*, 19, 490–498. doi: 10.3758/s13423-012-0239-5 22441958

[6] Wells G. L. (2020). Psychological science on eyewitness identification and its impact on police practices and policies. *American Psychologist*, 75, 1316–1329. doi: 10.1037/amp0000749 33382302

[7] Flowe H. (2011). An exploration of visual behaviour in eyewitness identification tests. *Applied Cognitive Psychology*, 25, 244–254. doi: 10.1002/acp.1670

[8] Flowe H. D., & Cottrell G. W. (2011). An examination of simultaneous lineup identification decision processes using eye tracking. *Applied Cognitive Psychology*, 25, 443–451. doi: 10.1002/acp.1711

[9] Mansour J., Lindsay R. C. L., Brewer N., & Munhall K. G. (2009). Characterizing visual behavior in a lineup task. *Applied Cognitive Psychology*, 23, 1012–1026. doi: 10.1002/acp.1570

[10] Brewer N., Gordon M., & Bond N. (2000). Effect of photoarray exposure duration on eyewitness identification accuracy and processing strategy. *Psychology*, *Crime*, *& Law*, 6, 21–32. doi: 10.1080/10683160008410829

[11] Charman S., & Wells G. L. (2007). Applied lineup theory. In Lindsay R. C. L., Ross D. F., Read J. D., & Toglia M. P. (Eds.), *The handbook of eyewitness psychology*, *Vol*. *2*: *Memory for people* (pp. 219–254). Lawrence Erlbaum Associates Publishers.

[12] Charman S. D., & Cahill B. S. (2012). Witnesses’ memories for lineup fillers postdicts their identification accuracy. *Journal of Applied Research in Memory and Cognition*, 1, 11–17. doi: 10.1016/j.jarmac.2011.08.001

[13] Wixted J. T., & Stretch V. (2004). In defense of the signal detection interpretation of remember/know judgments. *Psychonomic Bulletin and Review*, 11, 616–641. doi: 10.3758/bf03196616 15581116

[14] Brewer N. & Weber N. (2008). Eyewitness confidence and latency: Indices of memory processes not just markers of accuracy. *Applied Cognitive Psychology*, 22, 827–840. doi: 10.1002/acp.1486

[15] Brewer N., Caon A., Todd C. L. & Weber N. (2006). Eyewitness identification accuracy and response latency. *Law and Human Behavior*, 30, 31–50. doi: 10.1007/s10979-006-9002-7 16729207

[16] Dunning D., & Perretta S. (2002). Automaticity and eyewitness accuracy: A 10- to 12-second rule for distinguishing accurate from inaccurate positive identifications. *Journal of Applied Psychology*, 87, 951–962. doi: 10.1037/0021-9010.87.5.951 12395819

[17] Semmler C.A., Brewer N., & Wells G.L. (2004). Effects of postidentification feedback on eyewitness identification and nonidentification confidence. *Journal of Applied Psychology*, 89, 334–346. doi: 10.1037/0021-9010.89.2.334 15065979

[18] Sporer S. L. (1992). Post-dicting eyewitness accuracy-confidence, decision times and person descriptions of choosers and non-choosers. *European Journal of Social Psychology*, 22, 157–180. doi: 10.1002/ejsp.2420220205

[19] Weber N. & Brewer N. (2006). Positive versus negative face recognition decisions: Confidence, accuracy, and response latency. *Applied Cognitive Psychology*, 20, 17–31. doi: 10.1002/acp.1166

[20] Weber N., Brewer N., Wells G. L., Semmler C., & Keast A. (2004). Eyewitness identification accuracy and response latency: The unruly 10–12 second rule. *Journal of Experimental Psychology*: *Applied*, 10, 139–147. doi: 10.1037/1076-898X.10.3.139 15462616

[21] Lindsay R. C. L., & Wells G. L. (1985). Improving eyewitness identifications from lineups: Simultaneous versus sequential lineup presentation. *Journal of Applied Psychology*, 70, 556–564. doi: 10.1037/0021-9010.70.3.556

[22] Wilson B. M., Donnelly K., Christenfeld N., & Wixted J. T. (2019). Making sense of sequential lineups: An experimental and theoretical analysis of position effects. *Journal of Memory and Language*, 104, 108–125. doi: 10.1016/j.jml.2018.10.002

[23] Sporer S. L. (1993). Eyewitness identification accuracy, confidence and decision times in simultaneous and sequential lineups. *Journal of Applied Psychology*, 78, 22–33. doi: 10.1037/0021-9010.78.1.22

[24] Klobuchar A., Steblay N. K., & Caliguiri H. L. (2006). Improving eyewitness identifications: Hennepin County’s blind sequential lineup pilot project. *Cardozo Public Law*, *Policy*, *& Ethics Journal*, 4, 381–413.

[25] Fitzgerald R. J., & Price H. L. (2015). Eyewitness identification across the life span: A meta-analysis of age differences. *Psychological Bulletin*, 141, 1228–1265. doi: 10.1037/bul0000013 26011788

[26] Parker J. F., & Carranza L. E. (1989). Eyewitness testimony of children in target-present and target-absent lineups. *Law and Human Behavior*, 13, 133–149. doi: 10.1007/BF01055920

[27] Pozzulo J. D., & Lindsay R. C. L. (1998). Identification accuracy of children versus adults: A meta-analysis. *Law & Human Behavior*, 22, 549–570. doi: 10.1023/a:1025739514042 9833566

[28] Keast A., Brewer N., & Wells G. L. (2007). Children’s metacognitive judgments in an eyewitness identification task. *Journal of Experimental Child Psychology*, 97, 286–314. doi: 10.1016/j.jecp.2007.01.007 17512942

[29] Brewer N., Weber N., & Guerin N. (2020). Police lineups of the future? *American Psychologist*, 75, 76–91. doi: 10.1037/amp0000465 30998024

[30] Bruer K. C., Fitzgerald R. J., Price H. L., & Sauer J. D. (2017). How sure are you that this is the man you saw? Child witnesses can use confidence judgments to identify a target. *Law and Human Behavior*, 41, 541–555. doi: 10.1037/lhb0000260 28816467

[31] Colloff M. F., Seale-Carlisle T. M., Karoğlu N., Rockey J. C., Smith H. M. J., Smith L. L., et al. (2021). Perpetrator pose reinstatement during a lineup test increases discrimination accuracy. *Scientific Reports*, 11, 13830. doi: 10.1038/s41598-021-92509-0 34244529 PMC8271008

[32] Fitzgerald R. J., Price H. L., & Valentine T. (2018). Eyewitness identification: Live, photo, and video lineups. *Psychology*, *Public Policy*, *and Law*, 24, 30–325. doi: 10.1037/law0000164 30100702 PMC6078069

[33] Dalton G., Gawrylowicz J., Memon A., Milne R., Horry R., & Wright D. B. (2014). Public perceptions of identification procedures in the United Kingdom. *Policing*, 8, 35–42. doi: 10.1093/police/pat029

[34] Bailenson J. N., Davies A., Blascovich J., Beall A. C., McCall C., & Guadagno R. E. (2008). The effects of witness viewpoint distance, angle, and choice on eyewitness accuracy in police lineups conducted in immersive virtual environments. *Presence*: *Teleoperators and Virtual Environments*, 17, 242–255. doi: 10.1162/pres.17.3.242

[35] Oriet C., & Fitzgerald R. J. (2018). The single lineup paradigm: A new way to manipulate target presence in eyewitness identification experiments. *Law and Human Behavior*, 42, 1–12. doi: 10.1037/lhb0000272 29461076

[36] Fitzgerald, R. J., Rubínová, E., Ribbers, E., & Juncu, S. (under review). Eyewitness Lineup Identity (ELI) database: Crime videos and mugshots for eyewitness identification research. Manuscript submitted for publication. 10.3758/s13428-024-02585-z PMC11750919 39838182

[37] Smith A. M., Smalarz L., Ditchfield R., & Ayala N. T. (2021). Evaluating the claim that high confidence implies high accuracy in eyewitness identification. *Psychology*, *Public Policy*, *and Law*, 27, 479–491. doi: 10.1037/law0000324

[38] Malpass R. S. (1981). Effective size and defendant bias in eyewitness identification lineups. *Law and Human Behavior*, 5, 299–309. doi: 10.1007/BF01044945

[39] Fitzgerald R. J., Tredoux C. G., & Juncu S. (2023). Estimation of eyewitness error rates in fair and biased lineups. *Law and Human Behavior*, 4, 463–483. doi: 10.1037/lhb0000538 37471013

[40] Tredoux C. G. (1998). Statistical inference on measures of lineup fairness. *Law and Human Behavior*, 22, 217–237. doi: 10.1023/A:1025746220886

[41] Quigley-McBride A., & Wells G. L. (2021). Methodological considerations in eyewitness identification experiments. In Smith A. M., Toglia M., & Lampinen J. M. (Eds.), *Methods*, *measures*, *and theories in eyewitness identification tasks* (pp. 85–112). Routledge. doi: 10.4324/9781003138105-8

[42] Cohen J. (1988). *Statistical Power Analysis for the Behavioral Sciences* (2^nd^ ed.). Routledge.

[43] Ben-Shachar M. S., Lüdecke D., & Makowski D. (2020). effectsize: Estimation of effect size indices and standardized parameters. *Journal of Open Source Software*, 5(56), 2815. doi: 10.21105/joss.02815

[44] Mickes L. (2016). The effects of verbal descriptions on eyewitness memory: Implications for the real-world. *Journal of Applied Research in Memory and Cognition*, 5, 270–276. doi: 10.1016/j.jarmac.2016.07.003

[45] Price H. L., Bruer K. C., & Adkins M. C. (2020). Using machine learning analyses to explore relations between eyewitness lineup looking behaviors and suspect guilt. *Law and Human Behavior*, 44, 223–237. doi: 10.1037/lhb0000364 32105097

[46] Morgan C. A., Hazlett G., Doran A., Garrett S., Hoyt G., Thomas P., et al. (2004). Accuracy of eyewitness memory for persons encountered during exposure to highly intense stress. *International Journal of Law and Psychiatry*, 27, 265–79. doi: 10.1016/j.ijlp.2004.03.004 15177994

